# Heart Rate Fragmentation: A Symbolic Dynamical Approach

**DOI:** 10.3389/fphys.2017.00827

**Published:** 2017-11-14

**Authors:** Madalena D. Costa, Roger B. Davis, Ary L. Goldberger

**Affiliations:** ^1^Department of Medicine, Beth Israel Deaconess Medical Center, Margret and H. A. Rey Institute for Nonlinear Dynamics in Medicine, Harvard Medical School, Boston, MA, United States; ^2^Division of General Medicine and Primary Care, Department of Medicine, Beth Israel Deaconess Medical Center, Harvard Medical School, Boston, MA, United States

**Keywords:** aging, coronary artery disease, fragmentation, heart rate variability, symbolic dynamics, vagal tone

## Abstract

**Background:** We recently introduced the concept of heart rate fragmentation along with a set of metrics for its quantification. The term was coined to refer to an increase in the percentage of changes in heart rate acceleration sign, a dynamical marker of a type of anomalous variability. The effort was motivated by the observation that fragmentation, which is consistent with the breakdown of the neuroautonomic-electrophysiologic control system of the sino-atrial node, could confound traditional short-term analysis of heart rate variability.

**Objective:** The objectives of this study were to: (1) introduce a symbolic dynamical approach to the problem of quantifying heart rate fragmentation; (2) evaluate how the distribution of the different dynamical patterns (“words”) varied with the participants' age in a group of healthy subjects and patients with coronary artery disease (CAD); and (3) quantify the differences in the fragmentation patterns between the two sample populations.

**Methods:** The symbolic dynamical method employed here was based on a ternary map of the increment NN interval time series and on the analysis of the relative frequency of symbolic sequences (words) with a pre-defined set of features. We analyzed annotated, open-access Holter databases of healthy subjects and patients with CAD, provided by the University of Rochester Telemetric and Holter ECG Warehouse (THEW).

**Results:** The degree of fragmentation was significantly higher in older individuals than in their younger counterparts. However, the fragmentation patterns were different in the two sample populations. In healthy subjects, older age was significantly associated with a higher percentage of transitions from acceleration/deceleration to zero acceleration and *vice versa* (termed “soft” inflection points). In patients with CAD, older age was also significantly associated with higher percentages of frank reversals in heart rate acceleration (transitions from acceleration to deceleration and *vice versa*, termed “hard” inflection points). Compared to healthy subjects, patients with CAD had significantly higher percentages of soft and hard inflection points, an increased percentage of words with a high degree of fragmentation and a decreased percentage of words with a lower degree of fragmentation.

**Conclusion:** The symbolic dynamical method employed here was useful to probe the newly recognized property of heart rate fragmentation. The findings from these cross-sectional studies confirm that CAD and older age are associated with higher levels of heart rate fragmentation. Furthermore, fragmentation with healthy aging appears to be phenotypically different from fragmentation in the context of CAD.

*Words, words, words*. W. Shakespeare, Hamlet, Act 2, Scene 2

## 1. Introduction

Analysis of fluctuations in cardiac interbeat intervals, under the rubric of heart rate variability (HRV), continues to generate much interest as a uniquely accessible window into the complex network of regulatory mechanisms controlling the sino-atrial (SA) node (HRV, 1996; Billman, 2013). Particular emphasis has been placed on the analysis of short-term fluctuations, i.e., oscillatory patterns with cycle lengths ranging from approximately four to eight consecutive beats. Such fluctuations, termed respiratory sinus arrhythmia, are primarily ascribable to the coupling between heart rate and breathing, mediated by the parasympathetic (vagal) nervous system (Angelone and Coulter, 1964; Hirsch and Bishop, 1981).

**Figure 1:**
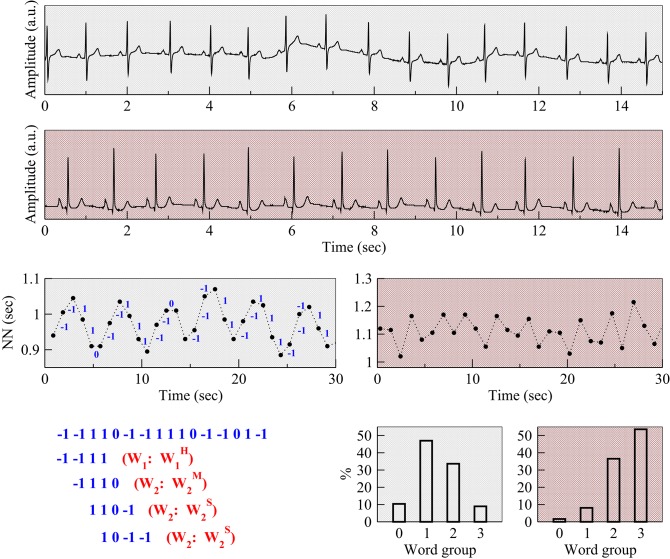
Examples of respiratory sinus arrhythmia and anomalous (fragmented) sinus rhythm. Electrocardiograms (Holter lead) from a healthy subject (first row) and a patient with coronary artery disease (CAD) (second row), both from the present study. Normal-to-normal (NN) sinus interval time series from the healthy subject (third row, left) and the patient with CAD (third row, right). The fluctuation patterns of the former time series are characteristic of phasic (respiratory) sinus arrhythmia, while that of the latter are indicative of an abnormal, non-phasic sinus arrhythmia (Costa et al., 2017). Positive and negative changes in the value of the NN intervals, corresponding to heart rate decelerations and accelerations were mapped to symbols “−1” and “1,” respectively. Symbol “0” is used to represent intervals in which heart rate did not change. To assist in visual comparisons, pale gray backgrounds are used for data from the healthy subject and light red for data from the patient with CAD, respectively. The symbolic mapping of the differences between consecutive NN intervals for the ECG of the healthy subject (first 16 intervals) along with the first four words that were derived from this sequence are shown on the bottom left. The first word “−1−111” contains one hard inflection point. It belongs to the group W_1_ and, more specifically, to the subgroup ${\text{W}}_{1}^{\text{H}}$. The following three words, “−1110,” “110−1,” and “10−1−1” contain two inflection points. Therefore, they belong to group W_2_. However, the first word (“−1110”) belongs to the subgroup ${\text{W}}_{2}^{\text{M}}$ since it contains one hard and one soft inflection point; the second (“110−1”) and the third (“10−1−1”) words belong to the subgroup ${\text{W}}_{2}^{\text{S}}$ since they present two soft inflection points. The panels on the bottom right show the percentage of words in each group for the healthy subject (left) and patient with CAD (right). Note a substantially higher percentage of fragmented words for the patient with CAD than for the healthy subject. The abbreviation “a.u.” stands for arbitrary units.

However, short-term fluctuations in heart rate are not always a marker of healthy cardiopulmonary interactions (Makikallio et al., 1999; Domitrovich and Stein, 2002; Stein, 2002; Wiklund et al., 2008; Costa et al., 2017) (Figure 1, first three rows). They may also be associated with abnormalities in the function of the neuroautonomic system, the SA node and other electrophysiologic components (Geiger and Goerner, 1945; Binkley et al., 1995; Jalife, 2013). Anomalous short-term variability is important for two major reasons: (1) it may confound the assessment of vagal tone modulation using conventional time and frequency domain HRV measures, leading to inflated estimates of “healthy” autonomic function in the elderly and especially in those with clinical or pre-clinical organic heart disease; and (2) its presence, itself, may be a novel dynamical biomarker of pathology and increased risk of adverse cardiovascular outcomes.

To help further address these issues, we (Costa et al., 2017) recently introduced the concept of *heart rate fragmentation*, along with a set of metrics to quantify this property. The underlying framework is based on the observation that sustained physiologic changes in heart rate cannot persist at frequencies higher than those at which the intact parasympathetic nervous system operates. Although the maximal physiologic response frequency is difficult to pinpoint, anticorrelated, beat-to-beat changes in heart rate, characterized by frequent changes from acceleration to deceleration and *vice versa*, are clearly atypical or frankly abnormal. The fragmentation indices that we introduced (Costa et al., 2017) quantify the density of this type of pattern. The assumption was that the systems manifesting the highest degree of fragmentation (loss of “fluency”) were the most pathologic ones.

We showed (Costa et al., 2017) that: (i) the degree of fragmentation of the NN and RR time series, derived from 24-h Holter monitoring, varied directly as a function of cross-sectional age in cohorts of healthy young to elderly male and female subjects in sinus rhythm and of those with coronary artery disease (CAD); (ii) the degree of fragmentation was significantly higher in patients with CAD than in healthy subjects, both in unadjusted models and in those adjusted for age and sex; and (iii) fragmentation indices outperformed standard time and frequency domain measures, as well as, widely used non-linear measures, in separating healthy subjects from patients with CAD.

To gain additional insight into the temporal structure of heart rate fragmentation, we now introduce a *symbolic dynamical* approach to the quantification of this property. In general, symbolic mapping deliberately reduces the overall information content of a signal. At the same time, it provides a useful way of highlighting certain features deemed of interest, while deemphasizing others. In heart rate fragmentation studies, examples of features of interest are the changes in heart rate acceleration sign, whereas “details” that one may choose to ignore are the magnitudes of those changes. In this study, the general hypotheses were that the degree of fragmentation, quantified by a set of variables derived from the symbolic dynamical analysis described below, would be: (1) higher in older subjects than in their younger counterparts, and (2) higher in patients with CAD than in healthy subjects. In addition, we sought to explore whether different symbolic “phenotypes” could help in distinguishing physiologic aging from aging in the context of overt organic heart disease.

## 2. Methods

### 2.1. Databases

We employed the same two long-term (~24-h) ECG ambulatory databases from the Intercity Digital Electrocardiogram Alliance (IDEAL) study previously analyzed (Costa et al., 2017). The deidentified recordings are made available via the University of Rochester Telemetric and Holter ECG Warehouse (THEW) archives (http://thew-project.org/databases.htm).

1. Healthy Subjects Database (THEW identification: E-HOL-03-0202-003) The database comprises 24-h Holter recordings from 202 ostensibly healthy subjects (102 males). The ECG signals were recorded at a sampling frequency of 200 Hz. Automated beat annotations were manually reviewed and adjudicated. We excluded 45 subjects with more than 2% non-sinus beats, 37 younger than 25 years old, 10 with BMI >30 Kg/m^2^ and one with <12 h of data. Overall, our analyses comprised 109 healthy adult subjects (60 male), age (median, 25th–75th) 40, 33–49 years.
2. Coronary Artery Disease Subjects Database (THEW identification E-HOL-03-0271-002).
  This database comprises 24-h Holter recordings from 271 patients (223 males). Subjects had an abnormal coronary angiogram (at least one vessel with luminal narrowing >75%) and either exercise-induced ischemia or a documented previous myocardial infarction. For our analysis, we also excluded 11 subjects whose Holter recordings contained ≥20% non-sinus beats and four with less than 12 h of data. Overall, we analyzed 256 subjects (208 male), age (median, 25th–75th): 60; 51–67 years; left ventricular ejection fraction 56.5%, 50–66.

As previously described Costa et al. (2017), presumed waking and sleeping periods were estimated as the six consecutive hours of highest and lowest heart rates, respectively. These periods were calculated from the NN interval time series using a 6-h moving average window shifted 15 min at a time. From the continuous ECG of each subject, the time series of the RR and NN intervals were derived. The former is the sequence of intervals between consecutive QRS complexes. The latter, is the subset of intervals between consecutive normal sinus to normal sinus QRS complexes.

### 2.2. Symbolic mapping and dynamical analysis

The original interbeat interval time series, {*s*_*i*_}, 1 ≤ *i* ≤ *N* (*N*, time series length) was mapped to a ternary symbolic sequence as follows: “−1” if ΔNN_*i*_ < 0, “0” if ΔNN_*i*_ = 0, and “1” if ΔNN_*i*_ > 0. Of note, since the ECG signals were sampled at 200 Hz, the resolution (τ) of both the NN interval and the increment time series was 5 ms (1/200 s). Taking the sampling frequency into consideration, the symbolic mapping rules can be alternatively written as: “1” if ΔNN_*i*_ ≤ −5 ms, “0” if −5 < ΔNN_*i*_ < 5 ms, and “−1” if ΔNN_*i*_ ≥ 5 ms. Next, the percentages of different segments of *l* consecutive symbols, *w*_*i*_ = (*s*_*i*_, *s*_*i*+1_, …, *s*_*i*+*l*−1_), 1 ≤ *i* ≤ *N*−*l*+1, termed “words,” were calculated. (With an alphabet of *n* symbols, the number of different words of length *l* is *n*^*l*^.) Words derived from the NN interval time series were termed NN words. Words derived from the RR interval time series were termed RR words.

**Figure 2:**
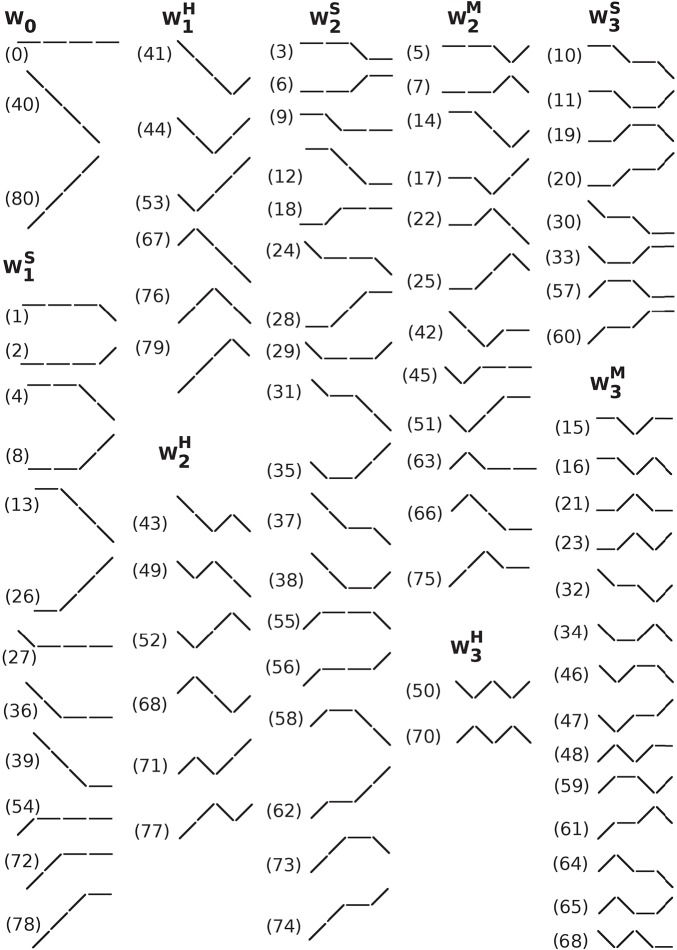
Schematic representation of all (#81) different words of length 4 with an alphabet of 3 symbols. The symbols “/”, “\”, and “−” represent heart rate acceleration, deceleration and no change, respectively. Words were grouped by the number and type of inflection points. The labels, 0–80, shown in parentheses, are the decimal value of the ternary representation of each pattern using the symbols “2” if ΔNN_*i*_ < 0, “1” if ΔNN_*i*_ > 0 and “0” if ΔNN_*i*_ = 0. For example, the label for the word comprising 4 consecutive accelerations, i.e., the word 2222, is 80 (= 2 × 3^3^+2 × 3^2^+2 × 3^1^+2 × 3^0^). Abbreviations: W, word subgroup. The subscript and superscript of W indicate, respectively, the number and the type of inflection points, hard (H), soft (S) or a combination of hard and soft (M, mixed) that the words in that subgroup contain.

Since we were interested in the analysis of short-term dynamical patterns occurring at the respiratory frequency, we chose a word length of four, which corresponds to time scales of approximately 3–5 s, depending on the heart rate. Subsequently, the words were grouped according to the number and type of transition between consecutive symbols (Figure 1). Reversals in heart rate acceleration (ΔNN_i_ × ΔNN_i+1_ < 0), i.e., transitions from symbol “1” to “−1” or *vice versa*, were termed hard (H) inflection points. Transitions to or from zero acceleration (ΔNN_i_ × ΔNN_i+1_ = 0, ΔNN_i_ ≠ ΔNN_i+1_), i.e., transitions from symbols “1” or “−1” to “0”, or *vice versa*, were termed soft (S) inflection points. The higher the number of inflection points in a word the more fragmented it was. Words of length four can contain no more than three inflection points. Word groups with only hard, only soft and a combination of hard and soft inflection points were, respectively, labeled ${\text{W}}_{j}^{\text{H}}$, ${\text{W}}_{j}^{\text{S}}$, and ${\text{W}}_{j}^{\text{M}}$ (where “M” stands for “mixed” and *j* indicates the number of inflection points). The word groups with more than one inflection point, W_*j*_ (2 ≤ *j* ≤ 3), for which the type of inflection point was not specified, comprised the words from subgroups ${\text{W}}_{j}^{\text{H}}$, ${\text{W}}_{j}^{\text{S}}$, and ${\text{W}}_{j}^{\text{M}}$. The word group W_1_ comprised the words from subgroups ${\text{W}}_{1}^{\text{H}}$ and ${\text{W}}_{1}^{\text{S}}$. Figure 2 shows a schematic representation of all the different words (*n* = 81).

Of note, to calculate the percentage of each NN word, two different denominators can be used: the total number of NN words and the total number of RR words. The former is not affected by the presence of ectopic beats, while the latter takes them into consideration. Here, the percentages of W_0_, W_*j*_, ${\text{W}}_{j}^{\text{H}}$, ${\text{W}}_{j}^{\text{S}}$, and ${\text{W}}_{j}^{\text{M}}$, 1 ≤ *j* < 3 were computed using the total number of NN words. In addition, we calculated the percentages of hard (soft) NN words that contained one, two and three hard (soft) inflection points. In these cases, the denominators were the total number of hard (soft) NN words with at least one inflection point. These word subgroups were labeled ${\text{W}}_{j}^{\text{H}*}$ (${\text{W}}_{j}^{\text{S}*}$). Thus, while ${\text{W}}_{j}^{\text{H}}$ (${\text{W}}_{j}^{\text{S}}$) represents the overall percentage of words with *j* hard (soft) inflection points, ${\text{W}}_{j}^{\text{H}*}$ (${\text{W}}_{j}^{\text{S}*}$) represents the percentage of hard (soft) words with *j* inflection points. They were calculated as follows: ${\text{W}}_{j}^{\text{H}*}$ = ${\text{W}}_{j}^{\text{H}}/\overset{3}{\underset{i=1}{\sum }}{\text{W}}_{i}^{\text{H}}$ (${\text{W}}_{j}^{\text{S}*}$ = ${\text{W}}_{j}^{\text{S}}/\overset{3}{\underset{i=1}{\sum }}{\text{W}}_{i}^{\text{S}}$).

The percentages of hard (PIP^H^) and soft (PIP^S^) inflection points were also computed. PIP^H^ and PIP^S^ are subcategories of the fragmentation index, PIP, previously introduced (Costa et al., 2017).

We analyzed how PIP^H^, PIP^S^ and the different group of words changed with the participants' age and with disease in unadjusted and adjusted [for age and sex, and age, sex and average NN interval (AVNN)] logistic models. Taking into consideration that heart rate fragmentation has been shown (Costa et al., 2017) to increase with cross-sectional age and with CAD in these databases, we specifically hypothesized that the percentages of words in groups W_0_ and W_0_ (least fragmented), would decrease with the participants' age and with disease, while the percentages of words in groups W_0_ and W_0_ (most fragmented), would increase, regardless of the type of inflection points.

### 2.3. Statistical analysis

Outcome variables were summarized by their median, 25th and 75th percentile values.

Linear regression models were used to quantify the dependence of each of the outcome variables (*y*: W_*j*_, ${\text{W}}_{j}^{\text{H}}$, ${\text{W}}_{j}^{\text{H}*}$, ${\text{W}}_{j}^{\text{S}}$, ${\text{W}}_{j}^{\text{S}*}$, ${\text{W}}_{j}^{\text{M}}$, PIP^H^, and PIP^S^) with the participants' age. These models included the interaction term between age and sample population to assess whether the regression slopes were the same in the two populations. In addition, these models included AVNN to control for the effects of this variable on each of the outcome variables (*y* = *c* + *β*_1_ × age + *β*_2_ × population + *β*_3_ × age × population + *β*_4_ × AVNN, where *c* is a constant). (The values of *β*_1_ and of *β*_1_+*β*_3_ along with their confidence intervals are provided in Table 1 for the groups of healthy subjects and those with CAD, respectively.) Statistical significance was set at a *p* < 0.05.

**Table 1:** Slope and [95%CI] of the association between each outcome measure and the participants' age for the group of healthy subjects and those with CAD, for the 24-h and putative awake and sleep periods.

**Variable**	**Healthy**			**CAD**		
	**24-h**	**Awake**	**Sleep**	**24-h**	**Awake**	**Sleep**
PIP^H^	0.036^†^ [−0.059, 0.131]	0.069^†^ [−0.041, 0.179]	−0.081 [−0.194, 0.032]	0.164 [0.096, 0.232]	0.271 [0.192, 0.350]	0.036 [−0.045, 0.117]
PIP^S^	0.262 [0.178, 0.345]	0.268^†^ [0.179, 0.357]	0.319^†^ [0.218, 0.420]	0.163 [0.104, 0.223]	0.144 [0.081, 0.208]	0.187 [0.115, 0.259]
W_0_	−0.118 [−0.160, −0.076]	−0.179 [−0.230, −0.127]	0.013^†^ [−0.027, 0.054]	−0.131 [−0.161, −0.101]	−0.183 [−0.220, −0.146]	−0.062 [−0.090, −0.033]
W_1_	−0.341 [−0.451, −0.232]	−0.330 [−0.439, −0.220]	−0.401 [−0.561, −0.241]	−0.317 [−0.396, −0.239]	−0.399 [−0.477, −0.321]	−0.206 [−0.321, −0.092]
${\text{W}}_{1}^{\text{H}}$	−0.365 [−0.478, −0.252]	−0.342 [−0.453, −0.232]	−0.463^†^ [−0.626, −0.301]	−0.301 [−0.382, −0.221]	−0.352 [−0.431, −0.273]	−0.228 [−0.344, −0.111]
${\text{W}}_{H}^{\text{1}*}$	−0.359 [−0.529, −0.189]	−0.396 [−0.580, −0.212]	−0.284 [−0.506, −0.062]	−0.413 [−0.535, −0.292]	−0.602 [−0.734, −0.471]	−0.205 [−0.364, −0.046]
${\text{W}}_{1}^{\text{S}}$	0.024^†^ [−0.005, 0.052]	0.013^†^ [−0.023, 0.048]	0.062^†^ [0.032, 0.092]	−0.016 [−0.037, 0.005]	−0.047 [−0.072, −0.021]	0.021 [0.000, 0.043]
${\text{W}}_{S}^{\text{1}*}$	−0.153 [−0.208, −0.098]	−0.178 [−0.238, −0.118]	−0.021 [−0.097, 0.055]	−0.173 [−0.212, −0.134]	−0.219 [−0.262, −0.176]	−0.071 [−0.126, −0.017]
W_2_	0.146^†^ [0.068, 0.223]	0.186 [0.107, 0.265]	0.051 [−0.073, 0.174]	0.045 [−0.011, 0.100]	0.102 [0.045, 0.158]	−0.070 [−0.159, 0.018]
${\text{W}}_{2}^{\text{H}}$	−0.046 [−0.159, 0.067]	0.002 [−0.110, 0.115]	−0.203 [−0.376, −0.030]	−0.020 [−0.101, 0.061]	0.079 [−0.002, 0.159]	−0.185 [−0.309, −0.062]
${\text{W}}_{H}^{\text{2}*}$	0.186 [0.063, 0.308]	0.234 [0.107, 0.361]	0.115 [−0.065, 0.295]	0.108 [0.020, 0.196]	0.225 [0.134, 0.316]	−0.046 [−0.175, 0.083]
${\text{W}}_{2}^{\text{S}}$	0.076^†^ [0.038, 0.114]	0.066^†^ [0.021, 0.110]	0.121^†^ [0.075, 0.168]	0.003 [−0.024, 0.030]	−0.035 [−0.067, −0.003]	0.049 [0.016, 0.082]
${\text{W}}_{S}^{\text{2}*}$	−0.033 [−0.095, 0.030]	−0.036 [−0.099, 0.027]	−0.161 [−0.252, −0.069]	−0.040 [−0.084.0.005]	−0.028 [−0.073, 0.017]	−0.104 [−0.169, −0.038]
${\text{W}}_{2}^{\text{M}}$	0.116^†^ [0.085, 0.146]	0.118^†^ [0.085, 0.150]	0.132^†^ [0.091, 0.174]	0.062 [0.040, 0.084]	0.058 [0.035, 0.081]	0.066 [0.037, 0.096]
W_3_	0.314 [0.193, 0.434]	0.322^†^ [0.194, 0.451]	0.337 [0.196, 0.478]	0.403 [0.317, 0.489]	0.480 [0.388, 0.572]	0.338 [0.237, 0.439]
${\text{W}}_{3}^{\text{H}}$	0.056^†^ [−0.019, 0.131]	0.043^†^ [−0.040, 0.125]	0.064 [−0.023, 0.151]	0.153 [0.099, 0.207]	0.196 [0.137, 0.255]	0.131 [0.068, 0.193]
${\text{W}}_{H}^{\text{3}*}$	0.173 [0.050, 0.297]	0.162^†^ [0.018, 0.307]	0.169 [0.038, 0.299]	0.305 [0.216, 0.393]	0.377 [0.274, 0.480]	0.251 [0.157, 0.344]
${\text{W}}_{3}^{\text{S}}$	0.052 [0.032, 0.072]	0.061 [0.038, 0.085]	0.050 [0.029, 0.072]	0.034 [0.020, 0.048]	0.034 [0.018, 0.051]	0.033 [0.018, 0.049]
${\text{W}}_{S}^{\text{3}*}$	0.186 [0.133, 0.239]	0.214 [0.156, 0.271]	0.181 [0.110, 0.252]	0.212 [0.174, 0.250]	0.247 [0.206, 0.288]	0.175 [0.124, 0.226]
${\text{W}}_{3}^{\text{M}}$	0.206 [0.138, 0.273]	0.218 [0.147, 0.290]	0.223 [0.144, 0.301]	0.216 [0.168, 0.264]	0.251 [0.199, 0.302]	0.174 [0.118, 0.230]

CAD, coronary artery disease; PIP, percentage of inflection points; W_j_, 0 ≤ j ≤ 3, group of words containing j inflection points; superscripts: H, hard inflection points; S, soft inflection points; M, mixed inflection points, i.e., a combination of hard and soft inflection points. Word groups for which the type of inflection point is not specified comprise words with all types of inflection points. The percentages of words in the groups $W_{j}^{H*}$ and $W_{j}^{S*}$ were calculated over the total number of NN words with only hard and only soft inflection points, respectively. The percentages of words in the other groups were calculated over the total number of NN words. Slope values marked with the symbol

† *are significantly different in the two sample populations*.

Logistic regression analysis was used to assess the relationships between presence of CAD and each of the outcome measures in unadjusted models, and models adjusted for age and sex, and age, sex, and AVNN. To facilitate comparisons among various outcome variables, we report normalized odds ratios (i.e., the odds ratio for a one standard deviation change in a given measure).

The area under the receiver operating characteristic (AUC) curve was used to assess the discrimination of each model. The likelihood ratio test was used to compare the fit of two nested models. All analyses were performed using raw measures.

## 3. Results

### 3.1. Relationship between heart rate fragmentation indices and participants' age

As previously reported in Costa et al. (2017), the overall percentage of inflection points (soft and hard combined) significantly increased with the participants' age in both healthy subjects and those with CAD. However, the percentages of only soft and only hard inflection points changed with the participants' age in different ways for each of the groups (Table 1). In healthy subjects, the percentage of soft inflection points significantly increased with the participants' age (slope of the regression line, [95% CI]: 0.26[0.18, 0.35] %/yr), while the percentage of hard inflection points did not (0.04[−0.06, 0.13] %/yr). In patients with CAD, both, the percentages of soft and hard inflection points increased with the participants' age at identical rates, 0.16[0.10, 0.22] %/yr and 0.16[0.10, 0.23] %/yr, respectively. These findings were consistent across all time periods with only one exception. During the putative sleep period, the increase in the percentage of hard inflection points did not reach statistical significance for those with CAD.

**Figure 3:**
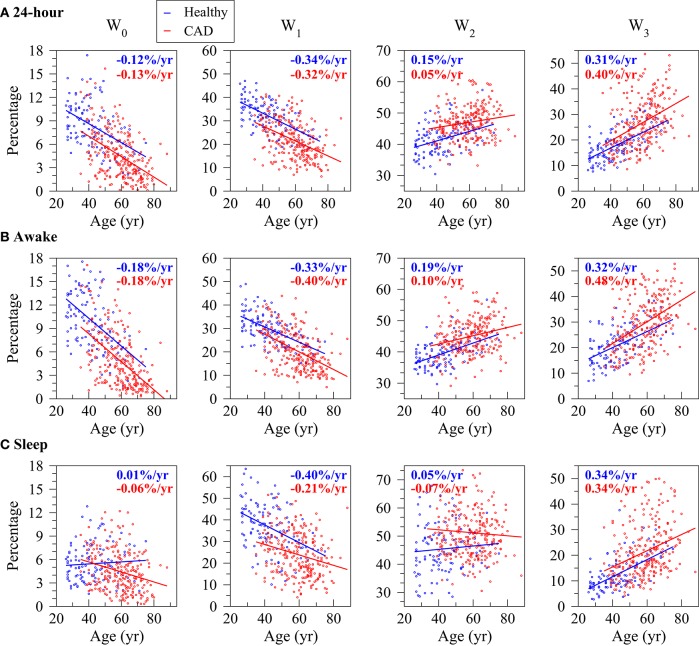
Relationship between the percentage of words with no inflection points (W_0_), one (W_0_), two (W_0_) and three (W_0_) inflection points and the participants' age for the healthy subjects (blue) and those with coronary artery disease (CAD, red) during the 24-h **(A)**, putative awake **(B)** and putative sleep **(C)** periods. Symbols and lines represent, respectively, word percentages for each subject and the regression lines derived from linear regression analyses controlled for the average NN interval. In each plot, the rates of change of the outcome variables per year of age for the healthy subjects and the patients with CAD are indicated in blue and red, respectively.

Overall, the percentage of words W_0_ and W_0_, i.e., the least fragmented (most “fluent”), decreased with the participants' age both in the groups of healthy subjects and those with CAD. All relationships were significant with the exception of the one with W_0_ during the putative sleep period for the healthy subjects. Complementarily, the percentage of words W_0_, the most fragmented (least “fluent”) significantly increased with the participants' age in both sample populations for all time periods (Figure 3 and Table 1). The percentage of words W_0_ that capture patterns of transitions between fluent and fragmented dynamics also tended to increase with the participants' age. However, only some of these relationships reached significance.

In both healthy subjects and patients with CAD, the word groups with the strongest association with the participants' age were W_1_, among the most fluent, and W_0_, among the most fragmented. In both cases, the magnitude of the rate of change was above 0.2%/yr (negative rate for W_1_ and positive rate for W_3_).

In general, the number of inflection points in a given word subgroup, not the type of inflection points (H, S or M) determined the directionality of the changes in its density with the participants' age. Among a total of 84 relationships (14 word subgroups, three time periods and two sample populations) only 7 did not change with the participants' age in the expected direction (Table 1); all except one of these relationships (${\text{W}}_{2}^{\text{S}}$ for the group of patients with CAD) were for the putative sleep period.

The most notable difference between healthy subjects and patients with CAD in the symbolic analysis related to the words with three hard inflection points (${\text{W}}_{3}^{\text{H}}$). While the percentage of these words did not significantly change with cross-sectional age in the group of healthy subjects for any time periods, it significantly increased in the group of patients with CAD, for all time periods, at rates varying between 0.13 and 0.20%/year.

### 3.2. Changes in heart rate fragmentation indices with coronary artery disease

A 1-year increase in age was associated with an increase of 14% in the odds of having CAD (odds ratio [95%CI]: 1.14[1.11, 1.17] <0.0001). The AUC for the model with age as the only covariate was 0.853. Male sex carried a 3.54-fold increase in the odds of CAD (3.54[2.17, 5.78], p < 0.0001). The AUC for the null model with age and sex as the sole independent variables was 0.882. The AUC for the null model with age, sex and AVNN was 0.910.

#### 3.2.1. Unadjusted analyses

Summary statistics of the calculated indices for the groups of healthy subjects and patients with CAD are presented in Table 2. In unadjusted analyses (Table 3), the percentage of hard inflection points, PIP^H^, was significantly higher in those with CAD than in healthy subjects for the three time periods. The percentage of soft inflection points, PIP^S^, showed a similar behavior. However, statistical significance was only reached for the putative sleep time.

**Table 2:** Measures of heart rate fragmentation/fluency in healthy subjects and those with coronary artery disease.

**Variable**	**Healthy**			**CAD**		
	**24-h**	**Awake**	**Sleep**	**24-h**	**Awake**	**Sleep**
	**Median, [25–75th]**	**Median, [25–75th]**	**Median, [25–75th]**	**Median, [25–75th]**	**Median, [25–75th]**	**Median, [25–75th]**
PIP^H^	36.9 [33.4–40.3]	33.8 [29.9–38.3]	41.2 [37.1–46.2]	42.9 [38.0–50.2]	41.2 [36.5–48.8]	45.5 [40.9–51.1]
PIP^S^	17.8 [13.9–22.8]	21.8 [15.6–27.6]	11.2 [8.44–15.8]	20.0 [15.4–24.4]	22.7 [17.5–28.9]	15.0 [11.2–20.5]
W_0_	8.33 [6.66–10.6]	9.91 [7.13–13.3]	5.08 [3.62–6.99]	4.35 [2.58–6.45]	4.22 [2.02–7.04]	4.21 [2.55–6.17]
W_1_	33.1 [27.7–36.8]	29.2 [26.1–33.7]	37.5 [30.2–45.0]	21.0 [15.6–27.2]	19.2 [13.7–26.7]	23.8 [15.9–32.0]
${\text{W}}_{1}^{\text{H}}$	27.8 [22.2–31.9]	21.9 [18.2–27.5]	34.7 [26.9–41.2]	16.9 [11.8–22.6]	14.4 [9.03–20.7]	20.3 [13.1–29.1]
${\text{W}}_{1}^{\text{H}*}$	50.7 [41.7–55.1]	47.9 [41.9–54.2]	50.3 [40.1–58.9]	31.1 [21.3–40.9]	29.6 [19.0–41.3]	33.3 [22.0–43.3]
${\text{W}}_{1}^{\text{S}}$	5.31 [3.66–7.05]	7.23 [4.55–9.23]	2.18 [1.37–3.43]	3.78 [2.48–5.36]	4.53 [2.81–6.75]	2.45 [1.40–3.55]
${\text{W}}_{1}^{\text{S}*}$	32.1 [29.8–34.0]	33.3 [30.8–35.7]	26.1 [22.8–29.3]	26.9 [23.7–29.5]	27.2 [23.9–30.5]	25.4 [21.4–27.9]
W_2_	41.4 [37.6–44.1]	39.4 [36.1–42.0]	44.3 [39.6–50.5]	46.6 [42.9–49.9]	44.8 [41.0–48.5]	49.1 [44.1–55.0]
${\text{W}}_{2}^{\text{H}}$	22.7 [18.5–28.0]	18.4 [14.5–24.7]	29.0 [23.1–40.7]	29.3 [23.4–35.1]	24.5 [18.4–31.7]	34.1 [27.2–42.5]
${\text{W}}_{2}^{\text{H}*}$	41.3 [36.4–47.4]	39.0 [35.3–44.6]	44.2 [35.5–53.6]	52.4 [46.6–57.7]	50.1 [43.5–56.0]	54.6 [46.1–62.9]
${\text{W}}_{2}^{\text{S}}$	9.08 [7.08–11.4]	10.8 [8.36–14.1]	5.73 [4.05–8.21]	8.21 [5.80–10.1]	9.21 [6.81–12.0]	6.05 [4.33–8.23]
${\text{W}}_{2}^{\text{S}*}$	55.2 [52.7–58.5]	53.4 [49.7–56.2]	64.2 [59.6–67.9]	56.5[53.9–59.9]	54.5 [51.6–58.7]	60.9 [57.1–65.3]
${\text{W}}_{2}^{\text{M}}$	7.91 [6.35–9.75]	8.56 [6.86–10.5]	6.63 [4.89–8.99]	8.74 [7.20–10.8]	9.61 [7.62–11.7]	8.09 [5.66–10.3]
W_3_	16.8 [13.3–20.4]	20.3 [15.1–25.1]	10.0 [7.99–15.8]	25.8 [19.4–32.6]	29.3 [21.2–36.8]	18.5 [13.9–26.9]
${\text{W}}_{3}^{\text{H}}$	4.44 [3.59–5.90]	5.02 [3.93–6.49]	3.08 [2.06–4.98]	7.36 [5.20–10.8]	7.89 [5.33–11.6]	5.43 [3.73–8.76]
${\text{W}}_{3}^{\text{H}*}$	8.81 [6.52–11.3]	11.0 [8.65–15.0]	4.39 [3.10–7.30]	14.2 [9.37–21.3]	16.9 [11.2–25.3]	9.58 [5.50–15.0]
${\text{W}}_{3}^{\text{S}}$	2.05 [1.23–3.25]	2.85 [1.50–4.27]	0.82 [0.47–1.54]	2.20 [1.34–3.29]	2.76 [1.71–4.52]	1.27 [0.71–2.37]
${\text{W}}_{3}^{\text{S}*}$	12.8 [10.3–15.1]	13.9 [10.1–16.4]	9.54 [7.72–12.6]	15.9 [13.3–19.7]	17.4 [13.9–20.8]	13.7 [10.4–17.3]
${\text{W}}_{3}^{\text{M}}$	9.58 [7.32–11.8]	12.1 [8.46–14.3]	5.96 [4.75–9.29]	14.2 [10.8–18.2]	15.9 [11.9–20.7]	10.6 [8.16–14.7]

Values are reported as median, 25th–75th percentiles. CAD, coronary artery disease; PIP, percentage of inflection points; W_j_, 0 ≤ j ≤ 3, group of words containing j inflection points; superscripts: H, hard inflection points; S, soft inflection points; M, mixed inflection points, i.e., a combination of hard and soft inflection points. Word groups for which the type of inflection point is not specified comprise words with all types of inflection points. The percentages of words in the groups $W_{j}^{H*}$ and $W_{j}^{S*}$ were calculated over the total number of NN words with only hard and only soft inflection points, respectively. The percentages of words in the other groups were calculated over the total number of NN words.

**Table 3:** Logistic regression analysis and area under the ROC curve for unadjusted models of CAD.

**Variable**	**24-h**		**Awake**		**Sleep**	
	**OR_n_ [95% CI]**	**AUC**	**OR_n_ [95% CI]**	**AUC**	**OR_n_ [95% CI]**	**AUC**
PIP^H^	3.66 [2.57–5.21]	0.765	3.97 [2.75–5.73]	0.776	1.79 [1.38–2.31]	0.652
PIP^S^	1.27 [1.00–1.61]	0.562	1.11 [0.89–1.39]	0.530	1.76 [1.34–2.32]	0.652
W_0_	0.27 [0.20–0.36]	0.820	0.26 [0.19–0.35]	0.832	0.70 [0.56–0.88]	0.609
W_1_	0.19 [0.14–0.28]	0.846	0.26 [0.19–0.36]	0.814	0.26 [0.19–0.36]	0.802
${\text{W}}_{1}^{\text{H}}$	0.27 [0.20–0.37]	0.802	0.39 [0.30–0.51]	0.747	0.27 [0.20–0.37]	0.798
${\text{W}}_{1}^{\text{H}*}$	0.17 [0.12–0.25]	0.852	0.21 [0.15–0.30]	0.837	0.31 [0.23–0.41]	0.778
${\text{W}}_{1}^{\text{S}}$	0.53 [0.42–0.67]	0.675	0.46 [0.36–0.59]	0.700	1.02 [0.81–1.28]	0.511
${\text{W}}_{1}^{\text{S}*}$	0.18 [0.12–0.26]	0.837	0.20 [0.14–0.30]	0.832	0.73 [0.57–0.93]	0.571
W_2_	3.68 [2.62–5.17]	0.783	3.79 [2.68–5.35]	0.794	1.85 [1.43–2.39]	0.660
${\text{W}}_{2}^{\text{H}}$	2.04 [1.56–2.67]	0.680	2.39 [1.78–3.21]	0.702	1.32 [1.04–1.66]	0.585
${\text{W}}_{2}^{\text{H}*}$	4.05 [2.90–5.65]	0.809	4.41 [3.08–6.31]	0.819	2.33 [1.79–3.04]	0.712
${\text{W}}_{2}^{\text{S}}$	0.67 [0.54–0.85]	0.605	0.58 [0.46–0.74]	0.636	1.06 [0.84–1.33]	0.516
${\text{W}}_{2}^{\text{S}*}$	1.31 [1.04–1.65]	0.582	1.42 [1.11–1.82]	0.596	0.65 [0.51–0.82]	0.630
${\text{W}}_{2}^{\text{M}}$	1.47 [1.15–1.88]	0.605	1.42 [1.12–1.81]	0.594	1.47 [1.15–1.87]	0.607
W_3_	4.32 [2.96–6.31]	0.790	3.23 [2.34–4.46]	0.759	4.56 [2.99–6.94]	0.790
${\text{W}}_{3}^{\text{H}}$	6.95 [3.80–12.7]	0.771	4.59 [2.74–7.70]	0.734	5.12 [2.83–9.25]	0.746
${\text{W}}_{3}^{\text{H}*}$	4.36 [2.81–6.77]	0.759	2.74 [1.95–3.86]	0.705	4.66 [2.79–7.79]	0.760
${\text{W}}_{3}^{\text{S}}$	1.13 [0.89–1.42]	0.526	1.01 [0.80–1.26]	0.505	1.61 [1.19–2.16]	0.624
${\text{W}}_{3}^{\text{S}*}$	2.79 [2.05–3.81]	0.728	2.47 [1.86–3.28]	0.714	2.83 [2.03–3.94]	0.731
${\text{W}}_{3}^{\text{M}}$	3.75 [2.60–5.42]	0.767	2.94 [2.14–4.05]	0.735	3.84 [2.61–5.66]	0.772

Values presented are normalized odds ratio (OR_n_), 95% confidence intervals (95% CI) and area under the receiver operating characteristic curve (AUC). CAD, coronary artery disease; PIP, percentage of inflection points; W_j_, 0 ≤ j ≤ 3, group of words containing j inflection points; superscripts: H, hard inflection points; S, soft inflection points; M, mixed inflection points, i.e., a combination of hard and soft inflection points. Word groups for which the type of inflection point is not specified comprise words with all types of inflection points. The percentage of word groups without “^*^” was calculated over the total number of NN words. The percentages of words in the groups $W_{j}^{H*}$ and $W_{j}^{S*}$ were calculated over the total number of NN words with only hard and only soft inflection points, respectively. The percentages of words in the other groups were calculated over the total number of NN words.

In these models, lower percentages of words without (W_0_) and with only one (W_0_) inflection point, and higher percentages of words with two (W_0_) and three (W_0_) inflection points were significantly associated with the presence of CAD, for all time periods. Similar results were obtained for the subgroups of hard (${\text{W}}_{j}^{\text{H}}$ and ${\text{W}}_{j}^{\text{H}*}$) and mixed (${\text{W}}_{j}^{\text{M}}$) words with any number of inflection points, for all time periods.

The percentages of words with one and two soft inflection points were significantly higher in healthy subjects than in patients with CAD for the 24-h and putative awake periods. For the sleep period, the differences were not significant. The percentage of soft words with three inflection points, the most fragmented of this class, tended to be higher in patients with CAD than in healthy subjects. However, significance was only reached for the sleep period.

#### 3.2.2. Analyses adjusted for age and sex

The percentage of hard inflection points, PIP^H^, remained positively associated with CAD in models adjusted for age and sex (Table 4) for the three time periods. The odds of CAD more than tripled, for each one-standard deviation increase in PIP^H^ during the 24-h and putative awake periods. For the putative sleep period, the odds doubled. The percentage of soft inflection points, PIP^S^ tended to be higher in healthy subjects than in those with CAD but the difference did not reach statistical significance for any of the time periods. Adding PIP^H^ to a model with only age and sex significantly improved its performance, whereas PIP^S^ did not.

**Table 4:** Logistic regression analysis and AUC for models of CAD adjusted for age and sex.

**Variable**	**24-h**			**Awake**			**Sleep**		
	**OR_n_ [95% CI]**	**AUC**	***p***	**OR_n_ [95% CI]**	**AUC**	***p***	**OR_n_ [95% CI]**	**AUC**	***p***
PIP^H^	3.24 [2.04–5.16]	0.911	1.55E-08	3.30 [2.03–5.35]	0.910	4.11E-08	2.03 [1.41–2.93]	0.897	4.38E-05
PIP^S^	0.78 [0.57–1.07]	0.885	1.23E-01	0.73 [0.53–1.01]	0.885	5.20E-02	0.96 [0.69–1.33]	0.882	8.01E-01
W_0_	0.37 [0.24–0.55]	0.907	1.80E-07	0.41 [0.28–0.62]	0.902	3.94E-06	0.56 [0.40–0.79]	0.894	5.48E-04
W_1_	0.39 [0.26–0.59]	0.900	2.56E-06	0.52 [0.35–0.77]	0.893	6.60E-04	0.42 [0.29–0.62]	0.898	2.17E-06
${\text{W}}_{1}^{\text{H}}$	0.56 [0.39–0.82]	0.890	1.91E-03	0.77 [0.55–1.08]	0.882	1.26E-01	0.46 [0.32–0.68]	0.894	2.44E-05
${\text{W}}_{1}^{\text{H}*}$	0.31 [0.20–0.48]	0.910	5.97E-09	0.37 [0.24–0.57]	0.905	8.62E-07	0.42 [0.29–0.60]	0.902	4.94E-07
${\text{W}}_{1}^{\text{S}}$	0.48 [0.34–0.66]	0.901	3.70E-06	0.47 [0.34–0.66]	0.901	5.33E-06	0.67 [0.49–0.91]	0.889	8.87E-03
${\text{W}}_{1}^{\text{S}*}$	0.26 [0.16–0.43]	0.910	5.26E-09	0.35 [0.22–0.56]	0.902	2.14E-06	0.58 [0.40–0.82]	0.890	1.76E-03
W_2_	2.79 [1.86–4.19]	0.906	3.34E-08	2.36 [1.58–3.53]	0.899	5.12E-06	2.05 [1.46–2.89]	0.896	9.13E-06
${\text{W}}_{2}^{\text{H}}$	2.33 [1.60–3.38]	0.904	1.43E-06	2.36 [1.59–3.50]	0.902	3.37E-06	1.78 [1.29–2.46]	0.894	2.55E-04
${\text{W}}_{2}^{\text{H}*}$	2.86 [1.95–4.21]	0.910	4.46E-09	2.78 [1.83–4.24]	0.907	4.41E-07	2.19 [1.56–3.06]	0.899	1.03E-06
${\text{W}}_{2}^{\text{S}}$	0.53 [0.38–0.73]	0.897	7.78E-05	0.52 [0.37–0.73]	0.897	6.33E-05	0.68 [0.50–0.93]	0.888	1.51E-02
${\text{W}}_{2}^{\text{S}*}$	1.62 [1.17–2.26]	0.889	3.17E-03	1.63 [1.16–2.29]	0.890	3.35E-03	1.08 [0.78–1.49]	0.882	6.35E-01
${\text{W}}_{2}^{\text{M}}$	0.92 [0.66–1.28]	0.882	6.19E-01	0.90 [0.64–1.26]	0.882	5.25E-01	0.96 [0.70–1.33]	0.882	8.09E-01
W_3_	1.75 [1.15–2.67]	0.889	6.03E-03	1.53 [1.04–2.24]	0.886	2.61E-02	1.78 [1.15–2.74]	0.888	5.71E-03
${\text{W}}_{3}^{\text{H}}$	2.69 [1.42–5.09]	0.895	2.99E-04	2.52 [1.38–4.61]	0.895	4.19E-04	1.96 [1.09–3.53]	0.887	9.21E-03
${\text{W}}_{3}^{\text{H}*}$	1.77 [1.10-2.86]	0.888	8.96E-03	1.48 [0.99–2.20]	0.885	4.17E-02	1.74 [1.05–2.90]	0.886	1.80E-02
${\text{W}}_{3}^{\text{S}}$	0.73 [0.54–1.01]	0.886	5.52E-02	0.69 [0.50–0.94]	0.887	1.69E-02	0.93 [0.67–1.31]	0.882	6.88E-01
${\text{W}}_{3}^{\text{S}*}$	1.30 [0.90–1.88]	0.882	1.54E-01	1.16 [0.81–1.66]	0.883	4.11E-01	1.54 [1.05–2.26]	0.886	2.03E-02
${\text{W}}_{3}^{\text{M}}$	1.51 [1.02–2.25]	0.886	3.23E-02	1.37 [0.95–1.98]	0.884	8.71E-02	1.63 [1.09–2.44]	0.887	1.26E-02

The analysis was performed using raw measures. Values presented are the normalized odds ratio (OR_n_) and the 95% confidence intervals (95% CI) for the variables listed in the header column, in models adjusted for age and sex; the area under the receiver operating characteristic curve (AUC) and the p value for the likelihood-ratio test of the null hypothesis that the addition of the HRV measure does not improve the fit of the model with age and sex alone. CAD, coronary artery disease; PIP, percentage of inflection points; W_j_, 0 ≤ j ≤ 3, group of words containing j inflection points; superscripts: H, hard inflection points; S, soft inflection points; M, mixed inflection points, i.e., a combination of hard and soft inflection points. Word groups for which the type of inflection point is not specified comprise words with all types of inflection points. The percentages of words in the groups $W_{j}^{H*}$ and $W_{j}^{S*}$ were calculated over the total number of NN words with only hard and only soft inflection points, respectively. The percentages of words in the other groups were calculated over the total number of NN words.

After adjusting for age and sex, the proportion of fragmented words, W_2_ and W_3_, remained positively associated with CAD (Table 4) for all time periods, while the proportion of fluent words, W_0_ and W_1_, remained negatively associated with CAD (Table 4) for all periods. Specifically, for the 24-h period, a one-standard deviation increase in W_2_ and W_3_ was associated with an increase of 180 and 75% in the odds of CAD, respectively. In addition, a one-standard deviation increase in W_0_ and W_1_ was associated with a drop of 63 and 61% in the odds of CAD, respectively. All word groups W_*j*_, 0 ≤ *j* ≤ 3, significantly improved the performance of a model with age and sex alone.

Hard word subgroups, ${\text{W}}_{j}^{\text{H}}$, and ${\text{W}}_{j}^{\text{H}*}$ changed with disease in the same way as the groups W_*j*_, (1 ≤ *j* ≤ 3). Specifically, ${\text{W}}_{1}^{\text{H}}$ and ${\text{W}}_{1}^{\text{H}*}$ were lower in those with CAD than in healthy subjects, for all time periods. All comparisons were statistically significant except the one with the variable ${\text{W}}_{1}^{\text{H}}$ for the putative awake period. In addition, ${\text{W}}_{2}^{\text{H}}$, ${\text{W}}_{3}^{\text{H}}$, ${\text{W}}_{2}^{\text{H}*}$ and ${\text{W}}_{3}^{\text{H}*}$ were significantly higher in those with CAD than in healthy subjects, for all time periods.

Soft word subgroups with one inflection point, ${\text{W}}_{1}^{\text{S}}$ and ${\text{W}}_{1}^{\text{S}*}$, changed with disease in the same way as the fluent, hard word subgroups. Specifically, they were more frequent in healthy subjects than in patients with CAD. In contrast, the percentages of soft words with two and three inflection points were lower in patients with CAD than healthy subjects. The comparison with ${\text{W}}_{2}^{\text{S}}$ were significant for all time periods. For ${\text{W}}_{3}^{\text{S}}$, only the comparison for the putative awake period reached significance. Mixed words with three inflection points were more discriminatory than those with two.

For the majority of cases (44 out of 54), adding a word group or subgroup to a model with age and sex significantly improved its performance. The exceptions were: ${\text{W}}_{2}^{\text{M}}$, ${\text{W}}_{3}^{\text{S}}$, and ${\text{W}}_{3}^{\text{S}*}$ for the 24-h period, ${\text{W}}_{1}^{H}$, ${\text{W}}_{2}^{\text{M}}$, ${\text{W}}_{3}^{\text{S}*}$ and ${\text{W}}_{3}^{M}$ for the putative awake time, and ${\text{W}}_{2}^{\text{S}*}$, ${\text{W}}_{2}^{\text{M}}$, and ${\text{W}}_{3}^{\text{S}}$ for the putative sleep time.

#### 3.2.3. Analyses adjusted for age, sex and AVNN

The major difference between the results of the analyses adjusted for age and sex and those adjusted for age, sex and AVNN, concerned the variable PIP^S^. When AVNN was added to the models, the differences in PIP^S^ between patients with CAD and healthy subjects became strongly significant: a one-standard deviation increase in PIP^S^ was associated with an increase in the odds of CAD ranging from 100 to 200%, for the different time periods.

In these models, PIP^H^ remained positively associated with CAD (Table 5) for the 24-h and putative awake periods, despite a decrease in the values of the odds ratio. However, for the sleep period, statistical significance was lost.

**Table 5:** Logistic regression analysis and AUC for models of CAD adjusted for age, sex and the average value of the NN intervals.

**Variable**	**24-h**			**Awake**			**Sleep**		
	**OR_n_ [95% CI]**	**AUC**	***p***	**OR_n_ [95% CI]**	**AUC**	***p***	**OR_n_ [95% CI]**	**AUC**	***p***
PIP^H^	2.09 [1.19–3.69]	0.914	7.67E-03	2.18 [1.26–3.77]	0.916	3.67E-03	1.29 [0.84– 1.98]	0.910	2.48E-01
PIP^S^	3.07 [1.74–5.42]	0.919	4.50E-05	2.61 [1.51–4.51]	0.918	3.62E-04	2.04 [1.29–3.21]	0.916	1.24E-03
W_0_	0.36 [0.23–0.55]	0.927	7.17E-07	0.37 [0.24–0.58]	0.925	2.16E-06	0.62 [0.44–0.88]	0.916	6.47E-03
W_1_	0.35 [0.22–0.56]	0.924	1.48E-06	0.33 [0.20–0.54]	0.927	1.10E-06	0.50 [0.33–0.74]	0.916	2.89E-04
${\text{W}}_{1}^{\text{H}}$	0.39 [0.25–0.60]	0.923	5.55E-06	0.38 [0.24–0.59]	0.924	5.76E-06	0.50 [0.34– 0.74]	0.915	2.77E-04
${\text{W}}_{1}^{\text{H}*}$	0.38 [0.24–0.59]	0.923	4.98E-06	0.34 [0.22–0.54]	0.928	5.52E-07	0.55 [0.37– 0.80]	0.915	1.46E-03
${\text{W}}_{1}^{\text{S}}$	0.82 [0.52–1.30]	0.910	3.98E-01	0.79 [0.50–1.24]	0.911	2.96E-01	1.09 [0.76– 1.58]	0.910	6.34E-01
${\text{W}}_{1}^{\text{S}*}$	0.32 [0.19–0.54]	0.923	5.05E-06	0.42 [0.25–0.69]	0.919	3.29E-04	0.70 [0.48– 1.02]	0.913	5.84E-02
W_2_	2.12 [1.36–3.31]	0.918	6.28E-04	1.89 [1.22–2.93]	0.915	3.06E-03	1.54 [1.06–2.23]	0.913	2.04E-02
${\text{W}}_{2}^{\text{H}}$	1.31 [0.78–2.21]	0.911	3.03E-01	1.31 [0.78–2.22]	0.911	3.04E-01	1.13 [0.77– 1.66]	0.910	5.40E-01
${\text{W}}_{2}^{\text{H}*}$	2.20 [1.46–3.34]	0.919	9.60E-05	2.26 [1.45–3.52]	0.920	1.40E-04	1.65 [1.15– 2.37]	0.915	5.49E-03
${\text{W}}_{2}^{\text{S}}$	1.11 [0.67–1.82]	0.911	6.89E-01	0.96 [0.60–1.52]	0.910	8.50E-01	1.27 [0.84– 1.92]	0.912	2.54E-01
${\text{W}}_{2}^{\text{S}*}$	0.71 [0.44–1.15]	0.911	1.63E-01	0.71 [0.44–1.14]	0.912	1.55E-01	0.62 [0.41– 0.94]	0.914	1.99E-02
${\text{W}}_{2}^{\text{M}}$	3.83 [2.18–6.72]	0.922	8.67E-07	3.37 [1.93–5.89]	0.920	7.00E-06	2.04 [1.31– 3.18]	0.917	1.09E-03
W_3_	3.78 [2.10–6.81]	0.926	4.16E-07	3.77 [2.15–6.61]	0.929	2.17E-07	2.32 [1.40–3.84]	0.916	3.38E-04
${\text{W}}_{3}^{\text{H}}$	2.15 [1.08–4.29]	0.913	1.88E-02	2.46 [1.24–4.89]	0.915	5.23E-03	1.51 [0.81– 2.83]	0.910	1.68E-01
${\text{W}}_{3}^{\text{H}*}$	2.44 [1.39–4.31]	0.916	4.67E-04	2.78 [1.60–4.83]	0.922	3.31E-05	1.82 [1.05– 3.17]	0.912	2.21E-02
${\text{W}}_{3}^{\text{S}}$	2.63 [1.48–4.67]	0.916	4.50E-04	1.97 [1.18–3.29]	0.914	7.47E-03	2.02 [1.23– 3.32]	0.914	2.31E-03
${\text{W}}_{3}^{\text{S}*}$	5.79 [2.97–11.1]	0.935	9.76E-10	3.90 [2.21–6.91]	0.929	2.74E-07	2.95 [1.79– 4.85]	0.923	2.22E-06
${\text{W}}_{3}^{\text{M}}$	4.46 [2.44–8.17]	0.931	1.31E-08	4.09 [2.34–7.13]	0.932	1.98E-08	2.51 [1.55– 4.06]	0.918	3.65E-05

The analysis was performed using raw measures. Values presented are the normalized odds ratio (OR_n_) and the 95% confidence intervals (95% CI) for the variables listed in the header column, in models adjusted for age, sex and the average value of the NN intervals (AVNN); the area under the receiver operating characteristic curve (AUC) and the p value for the likelihood-ratio test of the null hypothesis that the addition of the HRV measure does not improve the fit of the model with age and sex alone. CAD, coronary artery disease; PIP, percentage of inflection points; W_j_, 0 ≤ j ≤ 3, group of words containing j inflection points; superscripts: H, hard inflection points; S, soft inflection points; M, mixed inflection points, i.e., a combination of hard and soft inflection points. Word groups for which the type of inflection point is not specified comprise words with all types of inflection points. The percentages of words in the groups $W_{j}^{H*}$ and $W_{j}^{S*}$ were calculated over the total number of NN words with only hard and only soft inflection points, respectively. The percentages of words in the other groups were calculated over the total number of NN words.

For all of the time periods, fully adjusted models with word groups without inflection points, with one and three inflection points were more discriminatory than those with two inflection points (Table 5). Specifically, a one-standard deviation increase in the percentage of fluent words W_0_ and W_1_ was associated with a decrease in the odds of CAD ranging from 38 to 64% and from 50 to 67%, respectively. A one-standard deviation increase in the percentage of fragmented words W_3_ was associated with an increase in the odds of CAD ranging from 132 to 278%.

Of note, in these fully adjusted models, the number of inflection points, not their type (H, S or M), i.e., the degree of fragmentation of the words, determined the directionality of the effects in the odds of CAD. The majority of the word subgroups significantly improved the performance of a null model with age, sex and AVNN. Within group 1, the most discriminatory word subgroups were ${\text{W}}_{1}^{\text{H}}$ and ${\text{W}}_{1}^{\text{H}*}$. They appeared in significantly higher densities in healthy subjects than in patients with CAD, for all time periods. Within group 2, ${\text{W}}_{2}^{\text{H}*}$ and ${\text{W}}_{2}^{\text{M}}$ were the most discriminatory variables. For all time periods, significantly higher percentages of these words were observed in patients with CAD than in healthy subjects. Within group 3, all word subgroups were highly discriminatory of the two sample population. The only exception was ${\text{W}}_{3}^{\text{H}}$ during the putative sleep period. As expected, CAD was associated with a significant increase in the density of these words.

## 4. Discussion

Recently, we described a property of short-term HRV termed fragmentation and introduced a set of metrics to quantify this feature (Costa et al., 2017). The key marker of heart rate fragmentation is an overall increase in the frequency of changes in heart rate acceleration sign.

The purpose here was to further explore the property of heart rate fragmentation using symbolic dynamical analysis with the same databases previously studied. The findings were consistent with those reported in Costa et al. (2017), indicating an increase in heart rate fragmentation with the participants' age and with the presence of CAD. In addition, the symbolic analyses suggested a potentially important dynamical difference between aging in the healthy population and in those with coronary disease. This difference was not anticipated prior to this analysis.

Briefly, the notable findings of the cross-sectional study of heart rate fragmentation with the participants' age were that: (i) in the cohort of healthy subjects, the percentage of soft but not of hard inflection points significantly increased as a function of age; (ii) the percentages of both soft and hard inflection points significantly increased with the participants' age in the cohorts of subjects with CAD; (iii) overall, the density of fluent words tended to decrease and the density of the fragmented ones tended to increase with the participants' age in both populations, for all time periods (Figure 3); (iv) the percentage of words with three hard inflection points, the most fragmented, changed with age differently in the cohorts of healthy subjects and patients with CAD. For the latter, these words markedly increased for all time periods. For the former, the increases did not reach significance. The results from the symbolic analysis adjusted for age, sex and AVNN were consistent with the finding that the overall percentage of transitions from acceleration/deceleration to deceleration/acceleration did not change with the participants' age in the healthy group.

The key findings from the symbolic dynamical analysis comparing healthy subjects and those with CAD were that: (i) the percentages of fluent words, W_0_, W_1_, ${\text{W}}_{1}^{\text{H}*}$ were significantly higher in healthy subjects than in patients with CAD, for all time periods, in both unadjusted and adjusted models; (ii) the percentages of fragmented words, W_2_, ${\text{W}}_{2}^{\text{H}*}$, W_3_, and ${\text{W}}_{3}^{\text{H}*}$, were significantly lower in healthy subjects than in those with CAD, for all time periods and models; and (iii) although not all word subgroups were statistically different in the two sample populations for all time periods and models, importantly none of the fluent word subgroups, ${\text{W}}_{1}^{\text{H}}$, ${\text{W}}_{1}^{\text{S}}$, or ${\text{W}}_{1}^{\text{S}*}$ was significantly higher in patients with CAD than in healthy subjects for any time period or for any model. Similarly, none of the fragmented word subgroups with three inflection points, ${\text{W}}_{3}^{\text{H}}$, ${\text{W}}_{3}^{\text{H}*}$, ${\text{W}}_{3}^{\text{M}}$, ${\text{W}}_{3}^{\text{S}}$, and ${\text{W}}_{3}^{\text{S}*}$ was significantly higher in healthy subjects than in patients with CAD for any of the time periods in any model.

Overall, word subgroups with two inflection points were less discriminatory than the other ones, particularly in the fully adjusted models. This finding is not entirely surprising in light of the fact that words with two inflection points encode patterns that represent a transition between dynamical short-term fluency and fragmentation.

Qualitatively similar results to those presented here were obtained from the analysis of RR intervals time series (instead of NN) and words of length 5 (not presented here). Taken together these results robustly support the notion that heart rate fragmentation increases as a function of the participants' age and in the presence of overt CAD.

In this study, words without inflection points included segments of four consecutive accelerative, decelerative and zero acceleration intervals. Excluding the latter, i.e., the segments with no heart rate variability (neither fragmented nor fluent) from the word group W_0_, and quantifying their density separately, could potentially allow for a better characterization of a given study population, for example, one with chronic heart failure. In the present study, the results for the word group W_0_, including or excluding the word “0000,” were very similar. Therefore, we reported solely the results for which that word was included.

We wish to emphasize that the interpretation of the results for the word group W_0_ (with or without the inclusion of the word “0000”) can be dependent on the physiologic context. A deficit of these words is likely a consequence of a high degree of heart rate fragmentation. However, an excess is likely a consequence of long-term (above the normal respiratory frequency) trends in the data. These trends can be pathologic, as seen, for example, with sleep apnea syndromes (Guilleminault et al., 1984; Lipsitz et al., 1995; Guzik et al., 2013; Jiang et al., 2017), or physiologic, e.g., when associated with even mild bouts of exercise and recovery. The former conjecture is supported by the work of Guzik et al. (2013), who in a study of heart rate variability in subjects with various degrees of obstructive sleep apnea, found that an increased number of long (>5 intervals) deceleration and acceleration runs were most common in patients with severe sleep apnea.

Symbolic mapping of both the NN interval time series (Ravelo-Garcia et al., 2014; Cysarz et al., 2015) and of its increments have been used in many other studies, both in our laboratory (Yang et al., 2003; Costa et al., 2005, 2008) and others (Cysarz et al., 2000, 2015; Kantelhardt et al., 2002; Piskorski and Guzik, 2011; Guzik et al., 2012, 2013; Jiang et al., 2017). For example, Ashkenazy et al. (2001) used a binary map of the increment time series to analyze the correlation properties of the sign and magnitude heart rate time series of healthy subjects and patients with heart failure. For the shortest time scale explored, 6–16 NN intervals, they found that the dynamics of the sign time series of healthy subjects were closer to brown noise than those of patients with heart failure. This finding supports the hypothesis that long (>5 intervals) deceleration and acceleration runs are more common in healthy subjects than in patients with heart failure.

Guzik et al. (2012, 2013) and Piskorski and Guzik (2011) specifically analyzed the percentages of acceleration and deceleration runs of various lengths in a population of postinfarction patients. Overall, they found that decelerations runs of 2–10 intervals were significantly less frequent in non-survivors and used the runs of lengths 2, 4, and 8 to stratify all-cause mortality risk. The frequency of occurrence of runs of different lengths can be related to the concept of fragmentation. A higher percentage of short (<3) and a lower percentage of longer runs are expected in more fragmented than less fragmented time series. However, there is no direct correspondence between runs of a given length and a specific word. For example, runs of length 3 are necessarily part of the word group W_1_, but this word group also includes runs of lengths 1 and 2. Runs of length 2 are part of word groups W_1_ and W_2_; and runs of length 1 are part of all word groups but W_0_.

Cysarz et al. (2000, 2015) and Porta et al. (2007) used a binary map of the increment time series (“1” if ΔRR_*i*+1_ > RR_*i*_; “0” if ΔRR_*i*+1_ ≤ RR_*i*_) to analyze putative sympathetic/parasympathetic changes in neuroautonomic control under different conditions. However, for the types of fragmentation analyses proposed here, binary maps of heart rate increments are not recommended. In fact, if positive (or negative) and zero increments are mapped to the same symbol, soft inflection points are either “ignored” (when an accelerative interval is preceded or followed by an interval in which heart rate does not change), or “transformed” into hard inflection points (when a decelerative interval is preceded or followed by an interval in which heart rate does not change). Consequently, the word groups will contain words with different numbers of inflection points. For example, with the ternary map we used, the word group W_0_ contained only three words, specifically those labeled 0, 80, and 40 in Figure 2. However, with the binary map, this word group would also include the words 2, 8, 26, 54, 72, and 78 from ${\text{W}}_{1}^{\text{S}}$ (Figure 2), the words 6, 18, 28, 56, 62, and 74 from subgroup ${\text{W}}_{2}^{\text{S}}$, and the words 20 and 60, from subgroup ${\text{W}}_{3}^{\text{S}}$, with one, two and three soft inflection points, respectively. The same would be true for other word groups. Thus, the binary mapping of the NN interval time series does not preserve all the information necessary for assessing heart rate fragmentation.

Our analyses were based on the definition of acceleration/deceleration as a decrease (increase) in consecutive NN (RR) intervals of ≤ (≥) 5 ms. We could have chosen any multiple of 5 ms, but not a lower value, since, as mentioned in the Methods section, the ECG signals were recorded at 200 Hz. ECG signals recorded with a higher sampling frequency (SF) would permit other choices, specifically, any multiple of 1/SF. However, higher resolution/lower thresholds may not necessarily translate into an enhanced ability to discriminate different populations. In fact, the lower the threshold the more likely the results are to be affected by both biological and instrumental noise. On the other hand, the larger the threshold the higher the number of significant changes in acceleration/deceleration that will not be detected. Future studies will help determine an “optimal” range of thresholds for fragmentation analysis.

As noted, increased fragmentation under free-running conditions is not directly attributable to variations in sympathetic or parasympathetic activity (Costa et al., 2017). These autonomic effectors do not operate on fast enough time scales to account for sustained beat-to-beat changes in heart rate acceleration sign. However, such rapid heart rate acceleration changes have been noted with a variety of pathophysiologic alterations, including subtle premature supraventricular extrasystoles coming from the SA node itself (or from nearby areas), SA exit block variants, modulated sinus parasystole, and possibly mechanical atrial stretch effects (Friedman, 1956; Nazir and Lab, 1996). These conditions are most likely to occur with the breakdown of the sinus regulatory control, possibly due to inflammation or fibrosis at various anatomic sites (Ghiassian et al., 2016). In fact, the most common clinical settings of atrial fibrillation, which may represent an end stage of supraventricular fragmentation, are seen with aging and chronic heart disease, conditions in which vagal tone is usually diminished, SA node size is reduced and intercellular coupling may be impaired (Moghtadaei et al., 2016).

A notable but unanticipated finding of this study was the difference in the pattern of fragmentation seen in the cross-sectional analysis of older healthy subjects vs. those with organic heart disease (advanced atherosclerosis). Fragmentation in older healthy subjects was mostly due to the increase in the percentage of transitions from acceleration/deceleration to zero acceleration or *vice versa* (soft inflection points). Fragmentation in those with CAD, fragmentation was also due to the increase in the percentage of transitions from acceleration to deceleration or *vice versa* (hard inflection points).

Speculatively, the increase in hard inflection points with disease, i.e., the emergence of beat-to-beat reversals in heart rate acceleration, might also relate to higher degrees of fibrosis and inflammation, substrates for the development of conduction and/or pacemaker abnormalities. The increase in soft inflection points likely relate, in part, to the well-documented decrease in the variance of the NN interval high-frequency fluctuations with aging (Pikkujamsa et al., 1999). In fact, if the structure of the variability is sufficiently preserved (a sign of health), a decrease in the amplitude of the time series, would translate into an increase in the likelihood of having consecutive NN intervals with the same value, that is, of zero accelerations and thus of soft inflection points (assuming that the temporal resolution of the time series does not change).

Although a benign increase in heart rate fragmentation should be rare, it might arise with vagally induced prominent sinus bradycardia with SA Wenckebach, a condition sometimes seen in very healthy (athletic) young subjects. Future studies in well-characterized, larger databases, with outcome data related to incident atrial fibrillation and advanced sinus node disease, should also help ascertaining the translational value of the symbolic analysis of heart rate fragmentation proposed here and the utility of heart rate fragmentation as a quantifiable descriptor of HRV.

Finally, it may be of interest to explore the utility of the concept of dynamical fragmentation and adapt the symbolic dynamic analysis introduced here to the study of changes in repolarization parameters, such as those described under the heading of T wave alternans.

## 5. Conclusion

A symbolic dynamical approach to the analysis of heart rate increment time series in a cross-sectional study of healthy subjects and those with CAD, provides evidence supporting the conjecture that fragmentation increases with age and disease. In addition, our results suggest that fragmentation in ostensibly healthy aging is different from fragmentation in the context of overt disease. Future studies analyzing larger databases with outcome measures are needed to confirm these findings and to assess their translational value.

## Author contributions

MC and AG developed the fragmentation concept and symbolic approach to its quantification. RD directed the statistical analysis. All three authors contributed to the interpretation of the findings and worked collaboratively on the manuscript.

### Conflict of interest statement

The authors declare that the research was conducted in the absence of any commercial or financial relationships that could be construed as a potential conflict of interest.
